# Bee venom acupuncture for the treatment of chronic low back pain: study protocol for a randomized, double-blinded, sham-controlled trial

**DOI:** 10.1186/1745-6215-14-16

**Published:** 2013-01-14

**Authors:** Byung-Kwan Seo, Jun-Hwan Lee, Won-Suk Sung, Eun-Mo Song, Dae-Jean Jo

**Affiliations:** 1 Department of Acupuncture & Moxibustion, Kyung Hee University Hospital at Gangdong, #149 Sangil-Dong, Gangdong-Gu, Seoul, 134-727, Republic of Korea; 2 Department of Oriental Rehabilitation Medicine, Kyung Hee University Hospital at Gangdong, #149 Sangil-Dong, Gangdong-Gu, Seoul, 134-727, Republic of Korea; 3 Department of Neurosurgery, Kyung Hee University Hospital at Gangdong, #149 Sangil-Dong, Gangdong-Gu, Seoul, 134-727, Republic of Korea

**Keywords:** Bee venom acupuncture, Chronic low back pain

## Abstract

**Background:**

Chronic non-specific low back pain is the most common medical problem for which patients seek complementary and alternative medical treatment, including bee venom acupuncture. However, the effectiveness and safety of such treatments have not been fully established by randomized clinical trials. The aim of this study is to determine whether bee venom acupuncture is effective for improving pain intensity, functional status and quality of life of patients with chronic non-specific low back pain.

**Methods/design:**

This study is a randomized, double-blinded, sham-controlled clinical trial with two parallel arms. Fifty-four patients between 18 and 65 years of age with non-radicular chronic low back pain experiencing low back pain lasting for at least the previous three months and ≥4 points on a 10-cm visual analog scale for bothersomeness at the time of screening will be included in the study. Participants will be randomly allocated into the real or sham bee venom acupuncture groups and treated by the same protocol to minimize non-specific and placebo effects. Patients, assessors, acupuncturists and researchers who prepare the real or sham bee venom acupuncture experiments will be blinded to group allocation. All procedures, including the bee venom acupuncture increment protocol administered into predefined acupoints, are designed by a process of consensus with experts and previous researchers according to the Standards for Reporting Interventions in Clinical Trials of Acupuncture. Bothersomeness measured using a visual analogue scale will be the primary outcome. Back pain-related dysfunction, pain, quality of life, depressive symptoms and adverse experiences will be measured using the visual analogue scale for pain intensity, the Oswestry Disability Index, the EuroQol 5-Dimension, and the Beck’s Depression Inventory. These measures will be recorded at baseline and 1, 2, 3, 4, 8 and 12 weeks.

**Discussion:**

The results from this study will provide clinical evidence on the efficacy and safety of bee venom acupuncture in patients with chronic non-specific low back pain.

**Trial registration:**

This study is registered with the United States National Institutes of Health Clinical Trials Registry: NCT01491321

## Background

Chronic non-specific low back pain (CLBP) is a common medical problem considered as a multifactorial disorder in which musculoskeletal pain and psychosocial factors interact with each other [1,2]. The socioeconomic impact of CLBP is related to its greater comorbidities and more frequent prescriptions of pharmacotherapies and other adjunctive medications associated with these painful conditions [3]. Despite of variable accessibility to conventional treatments, patients with low back pain (LBP) have increasingly been using complementary and alternative medicine to alleviate their symptoms [4]. The tendency towards the use of complementary and alternative medicine in CLBP may reflect the deficits and unfulfilled patient expectations in conventional medical treatment [5]. Therapeutic modalities of complementary and alternative medicine, including acupuncture, herbal medicine, thermal therapy and spinal manipulative therapy, have been used in patients with CLBP [5,6] but evidence of effectiveness has not been fully established.

Bee venom acupuncture (BVA) involves injecting purified and diluted bee venom into acupoints [7]. BVA exhibits several pharmacological actions, including analgesic, anti-inflammatory, anti-arthritic, and anti-cancer effects through multiple mechanisms, such as activation of the central inhibitory and excitatory systems and modulation of the immune system [8]. The analgesic effects of BVA have been reported in animal experiments [9,10] and in the clinic [7,11]. Researchers have found that BVA could be a therapeutic option for alleviating LBP [7,12]. Recently, a cohort study revealed that the bee venom integrative package, which comprises herbal medicines, acupuncture and spinal manipulation, is effective in the treatment of LBP with leg pain [13]. However, there has been relatively little evidence in clinical trials on BVA to treat CLBP, especially rigorous randomized controlled clinical trials on the efficacy of BVA. Therefore, a rigorous randomized controlled trial is needed to develop clinical indications and a manual for the optimal practical guidelines of BVA.

### Aims

The purpose of this study is to determine whether BVA is effective at improving pain intensity, functional status and quality of life in patients with CLBP.

## Methods/design

### Design

This study is a randomized, double-blinded, sham-controlled clinical trial with two parallel arms. We intend to compare the effects of BVA and pharmacotherapy with sham-BVA and pharmacotherapy in patients with CLBP. The trial is registered with the U.S. National Institutes of Health Clinical Trials registry and is approved by the Institutional Review Board of Kyung Hee University Hospital at Gangdong (KHUHGD). The outcome assessment and the statistical analyses are performed by professionals who are blinded to the assignment of patients to the real or sham BVA group.

### Participants

Patients with non-radicular chronic LBP will be recruited with a target sample size of 54 participants.

Inclusion criteria:

Being between 18 and 65 years of age

Experiencing LBP lasting for at least the previous three months

Scoring ≥4 points on a 10-cm visual analog scale (VAS) for bothersomeness of LBP at the time of screening

Exhibiting no abnormalities on neurological examination (for example, lumbosacral nerve function, deep tendon reflexes, plantar response, voluntary muscle activation and sensory function)

Having non-specific, uncomplicated LBP, that is, International Classification of Diseases-10 codes:

M513 Other specified intervertebral disc degeneration

M545 Low back pain

M548 Other dorsalgia

M549 Dorsalgia, unspecified

S335 Sprain and strain of lumbar spine

S337 Sprain and strain of other and unspecified parts of the lumbar spine and pelvis

S336 Sprain and strain of sacroiliac joint

Agreeing to participate and signing informed consent

Exclusion criteria:

Radicular pain

Serious spinal disorders, including malignancy, vertebral fracture, spinal infection and inflammatory spondylitis

Other chronic diseases that could affect or interfere with the therapeutic outcomes, including cardiovascular disease, diabetic neuropathy, active hepatitis, fibromyalgia, rheumatoid arthritis, dementia and epilepsy

Previous spinal surgery or scheduled procedures during the study

Painful conditions induced by traffic accidents

Chief musculoskeletal pain other than back pain

Conditions where BVA might not be safe, including clotting disorders, administration of an anticoagulant agent, pregnancy and seizure disorders

Documented hypersensitive reactions to previous BVA treatments, bee stings or insect bites

Severe psychiatric or psychological disorders

Current use of corticosteroids, narcotics, muscle relaxants or herbal medicines to treat LBP or any medication considered inappropriate by the investigator

Pending lawsuit or receipt of compensation because of LBP

Refusal to participate in the trial or provide informed consent

Inability to read and write in the Korean language

### Recruitment

**Figure 1:**
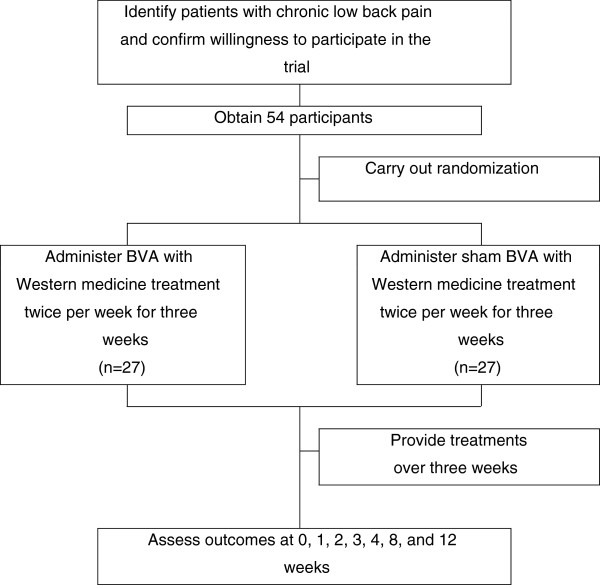
**Study sequence.** Process of recruitment, randomization to treatment and outcome assessment.

Participants will be recruited through advertisements in local newspapers, on hospital websites and on bulletin boards. If patients are interested in participation, they will be asked to answer screening questions to determine their eligibility. If eligible, they will be guided through the informed consent process. After written consent is obtained, a study researcher will administer the baseline questionnaire then randomly allocate the participants into the real or sham BVA group. After randomization, the interviewer will schedule the treatment procedure. All recruit procedures will be recorded in a log file (Figure 1).

### Randomization and blinding procedure

Randomization will be undertaken through a computerized software randomization program by an independent statistician who is concealed with respect to the patients. Randomization will be performed only after a participant is confirmed to be eligible and written informed consent has been obtained. Participant details will be recorded, and the treatment arm and randomization number will be allocated to the patients; both of these are included in the participants’ hospital files. The randomization form will be completed and returned to the principal investigator.

As a double-blinded randomized controlled trial, the researcher performing the outcome measure assessments will be blinded to the patient’s treatment allocation. Furthermore, the people who administer treatments will be blinded as to whether the patient receives real or sham BVA, and the pharmacopunctures will be prepared by independent researchers.

### Education of acupuncturists

Licensed Korean medical doctors (KMDs) who are specialized in acupuncture and moxibustion or oriental rehabilitation medicine will take an educational course to ensure their strict adherence to the study protocol and familiarity with administering study treatments; all participating KMDs will undergo intensive and customized training for a full understanding of the BVA increment protocol, including details such as acupuncture points and weekly dose increments of pharmacopuncture.

### Description of all interventions and comparisons

#### Treatment details

Both the real and sham BVA groups will receive a total of six pharmacopuncture sessions over three weeks. In the BVA group, 26-gauge sterile disposable syringes (Noksipja, Seoul, Korea) containing bee venom diluted with normal saline (0.9% NaCl) will be used, whereas normal saline without bee venom with the same dose will be used in the sham BVA group. A skin hypersensitivity test will be performed on all the patients at LI 11 acupoints with subcutaneous injections of 0.05 ml of 1:20,000 bee venom preparation. Local swelling over 10 mm in diameter or redness over 20 mm in diameter will be considered positive reactions, and these patients will be excluded from this study. At the first pharmacopuncture session, all participants will receive a copy of the *Exercise Manual for Patients with Low Back Pain* from the spine center of KHUHGD and will be encouraged to exercise according to the manual during the entire treatment period. As a rescue therapy to minimize the risk of disease deterioration during the trial period, all the participants will be prescribed loxonin (Loxoprofen, 60 mg/tablet; Dong Wha Pharm Co., Ltd., Seoul, Korea) per os one tablet three times a day throughout the treatment period.

#### Bee venom treatment protocol

BVA will be prepared as dried bee venom powder (Yoomil Garden, Hwasun, Korea) then diluted and filtered in normal saline (1:20,000) at the Traditional Korean Medical Pharmacy at KHUHGD. After sterile skin preparation, patients from both real and sham BVA group will be subcutaneously injected perpendicularly at a depth of 0.5 cm to 1.0 cm with the patient lying in a prone position. Pharmacopuncture will be prepared by a predefined weekly incremental protocol as follows; 0.2 ml for the first week, 0.4 ml for the second week and 0.8 ml for the third week. Patients in the sham BVA group will be injected with the same volume as those in the BVA group. The predefined points are carefully selected by a process of consensus among participating KMDs who are all experienced in the treatment of LBP. Acupuncturists can select up to 10 points from predefined acupoints as a reflection of daily practice, in which the acupuncture points are varied based upon changes of patient symptoms or pain (Table 1).

**Table 1:** Bee venom acupuncture treatment and sham treatment protocol

	**Item**	**Details**
1. Acupuncture rationale	(a) Style of acupuncture	Traditional Korean medicine theory
	(b) Reasoning for treatment provided (based on historical context, literature sources and consensus methods)	Textbook on acupuncture and moxibustion-related articles (published trials) [14]
	(c) Extent to which treatment was varied	Within 10 predefined acupoints
2. Details of needling	(a) Number of needle insertions per patient per session	Within 10 acupoints
	(b) Names (or location if no standard name) of points used (uni/bilateral)	Shenshu (BL23), Qihaishu (BL24), Dachangshu (BL25), Huantiao (GB30), Yaoyangguan (GV3), Mingmen (GV4), Xuanshu (GV5)
	(c) Depth of insertion	5 to 10 mm (subcutaneous)
	(d) Response sought (for example, *de qi* or muscle twitch response)	Penetrating, sharp, aching and painful sensations when penetrating the skin. Spreading and lumpish sensation around the injection site. Skin stimulation by bee venom in BVA group.
	(e) Needle stimulation (for example, manual, electrical)	Pharmacopuncture
	(f) Needle-retention time	None
	(g) Needle type (diameter, length and manufacturer or material)	1.0 ml disposable syringe (26-gauge needle) produced by Green Cross Medical Equipment (Seoul, Korea)
3. Treatment regimen	(a) Number of treatment sessions	Six
	(b) Frequency and duration of treatment sessions	Twice a week for three weeks
4. Other components of treatment	(a) Details of other interventions administered to the acupuncture group	Brochure with information about chronic low back pain, lifestyle advice
	(b) Setting and context of treatment, including instructions to practitioners and information and explanations to patients	Independent researcher counseling regarding treatment, lifestyle management of low back pain
5. Practitioner background	(a) Description of participating acupuncturists	Korean medical doctors who are specialists of acupuncture and moxibustion or oriental rehabilitation medicine, with more than three years of clinical experience under supervision by a specialist
6. Control or comparator interventions	(a) Rationale for the control or comparator in the context of the research question with sources that justify this choice	See [15]
		See [16]
		See [17]
	(b) Precise description of the control or comparator if sham acupuncture	**Double blind:** In the sham BVA group, normal saline was used instead of BVA. Both real and sham BVA were subcutaneously injected into predefined acupoints through identical manipulation techniques and the assessor, acupuncturist and patients were uninformed about allocation.
		**Explanations given to patients:** Explanation of real or sham BVA was given to patients before randomization.
		**Details of sham BVA:** Acupuncture points used, needle type, depths of insertion, responses, needle stimulation and needle retention time were identical in each group. The only difference was that normal saline was used for injection.
		**Injection and increment protocol:** The BVA group was prepared by a predefined weekly increment protocol as follows: 0.2 ml for the first week, 0.4 ml for the second week and 0.8 ml for the third week. The sham BVA group was injected with normal saline at the same volume as the BVA group. The predefined points were carefully selected by a process of consensus with the participating Korean medical doctor.

BVA: bee venom acupuncture.

### Data collection

A series of measurements to assess back pain-related dysfunction, pain, quality of life and adverse experiences will be collected at baseline and week 1, 2, 3, 4, 8 and 12. At the screening visit, patients will be asked to fill out a questionnaire regarding their sociodemographic characteristics, including age, gender, marital status, residence, occupation and education level. A medical history will be also taken before their physical check-ups, and these factors will determine their eligibility for our study. Participation in can be ended at any stage if the patient refuses to continue or in the presence of significant clinical deterioration, as determined by the attending KMD researchers. For those participants who will be lost to follow-up and who drop out, intention-to-treat analysis will be applied to the existing data (Table 2).

**Table 2:** Schedule for data collection: outcome measures per visit

**Measures**	**Baseline (Week 0)**	**Week 1**	**Week 2**	**Week 3**	**Week 4**	**Week 8**	**Week 12**
Sociodemographic characteristics	x						
Back pain history	x						
X-ray	x						
Visual analogue scale for bothersomeness of low back pain	x	x	x	x	x	x	x
Visual analogue scale for pain intensity of low back pain	x	x	x	x	x	x	x
Oswestry Disability Index	x	x	x	x	x	x	x
EuroQol-5D	x	x	x	x	x	x	x
Beck's Depression Inventory	x	x	x	x	x	x	x
Adverse experiences^a^		x	x	x	x		x
Credibility test^b^	x			x			

a. Adverse experiences : Any adverse experiences at any visit during treatment sessions and 12 weeks will be monitored. The research team will review all trial protocols, monitor patient safety and investigate any adverse events. b. Credibility test : The credibility of the real and sham treatments will be assessed using the validated Korean version of the credibility test.

#### Primary outcome measurements

Bothersomeness from LBP will be assessed using the 10-cm VAS for bothersomeness of LBP [18]. To evaluate the clinical severity and impact on activities of daily life in patients with CLBP, the VAS for bothersomeness is selected as a primary outcome measurement. Using the 10-cm VAS (0, absence of bothersomeness; 10, the worst bothersomeness imaginable), the patient will be asked to report the degree of bothersomeness from LBP within the past week. Bothersomeness of LBP will be measured at every single visit at baseline and 1-, 2-, 3-, 4-, 8- and 12-week follow-ups after beginning treatment. The primary end point is the third week follow-up, which marks the end of the six pharmacopuncture sessions.

#### Secondary outcome measurements

VAS for pain intensity: The pain intensity of LBP will be assessed using the 10-cm VAS for LBP [19,20]. This inventory is a fast and straightforward method for evaluating the subjective degree of pain. Pain intensity will be measured in the same fashion as VAS for bothersomeness (0, absence of pain; 10, the worst pain imaginable) at every single visit at baseline and 1-, 2-, 3-, 4-, 8- and 12-week follow-up visits after beginning treatment.

The Oswestry Disability Index (ODI): Back pain related dysfunction will be assessed using the ODI [21]. The ODI contains 10 questions about daily activities, including inventories of pain intensity, personal care, lifting, walking, sitting, standing, sleeping, sexual life, social life and traveling. Each question is rated on a scale from 0 to 5 points; the lower the score, the less disabled the person is by the pain. The validated Korean Version of the ODI [22] will be administered at baseline and 1-, 2-, 3-, 4-, 8- and 12-week follow-up visits after beginning treatment.

The EuroQol 5-Dimension (EQ-5D): Quality of life for patients with LBP will be assessed using the Korean version of the EQ-5D [23,24]. The EQ-5D includes generic questions about quality of life as it relates to personal health. This inventory has two parts. In the first part, tthe patient evaluates his or her health state with respect to five dimensions in a descriptive way: mobility, personal care, daily activities, pain or discomfort, and anxiety or depression. Each dimension is scored on a scale from 1 to 3; the lower the score, the better the state of health of the test taker. In the second part, the patient rates his or her overall state of health on the day the questionnaire is completed using a VAS scale from 0, which is the worst imaginable state of health, to 100, the best possible state of health. The two scores are complementary. The EQ-5D has an index of reference value of possible health profiles ranging from a value of 1 (the best state of health) to 0 (death). The EQ-5D will be administered at baseline and 1-, 2-, 3-, 4-, 8- and 12-week follow-up visits after treatment begins.

Beck’s Depression Inventory (BDI): Depressive symptoms from LBP will be assessed by the Korean version of BDI [25,26]. The BDI includes 21 items and is a self-administered questionnaire. Each item has a response format ranging from 0 to 3, giving a theoretical maximum score of 63. The BDI will be administered at baseline and 1-, 2-, 3-, 4-, 8- and 12-week follow-ups after beginning treatment.

Credibility test: The credibility of the real and sham treatments will be assessed using the validated Korean version of the credibility test at baseline and again at the end of treatment [27]. The participants will rate the credibility of the treatment by answering four questions on a numeric rating scale, with 0 being not at all and 6 for maximal agreement:

1. improvement expected;

2. recommendation to others;

3. treatment logical;

4. effective also for other diseases.

#### Safety

Any adverse experiences at any visit during the six treatment sessions will be monitored. The research team will review all trial protocols, monitor patient safety and investigate any adverse events, which are defined as treatment-related experiences.

### Withdrawal and dropout

Participation will be ended at any stage if the patient refuses to continue, withdraws consent, violates inclusion or exclusion criteria or the trial protocol, or completes less than four treatment sessions as determined by the attending KMD researchers. The trial will be stopped if the principle investigator believes that there are unacceptable risks of serious adverse events.

### Sample size determination

We have performed sample size calculations for CLBP according to another acupuncture study on the same disease using the same primary outcome measure because no such BVA research has been reported [28]. The expected mean difference and common standard deviation between the two groups are estimated (mean difference = 1.5, SD = 2.73) according to that former acupuncture research [28] on CLBP using the 10-cm VAS as a primary outcome measure. For two arms (control-sham BVA and experimental-BVA), we consider the two-sample *t*-test model. Twenty-seven participants will be required per intervention group to achieve 0.80 power (1-β) at the 0.05 α level after adjusting for a 20% attrition rate.

### Data analysis

The statistical analysis will be performed in the principle of intention-to-treat analysis and per-protocol analysis. For the intention-to-treat analysis, data will be processed with the last observation carried forward method.

A homogeneity test of the baseline characteristics will be performed on both demographical and clinical data using a two-sample *t*-test for quantitative data and a chi-square test for qualitative data. The two-sample *t*-test will be used for the VAS to evaluate the bothersomeness of LBP at baseline and again at four weeks (primary endpoint) for the comparison of the two groups and to determine differences from baseline. For each outcome variable, an analysis of covariance will be performed on the data to adjust the baseline characteristics. Trends over time and time-by-treatment interactions will be explored using a repeated-measures analysis of variance. A chi-square test or a Fisher’s exact test will be performed to determine the difference between groups and the adverse effects, which will be recorded and described as frequency and percentage. All statistical analyses will be carried out with the Statistical Package for the Social Sciences (SPSS, Inc., Chicago, IL, USA) for Windows version 18.0, and a significance level of 0.05 will be used.

### Data handling

Investigators will enter the information required by the protocol into the case report forms. Non-obvious errors or omissions will be put into data query forms, which will be used for the researchers’ workshop. The data will be gathered and summarized with respect to demographic baseline characteristics, effectiveness and safety observations.

## Discussion

The results from this study will determine the efficacy and safety of BVA on CLBP as an adjunctive treatment to pharmacotherapy that reflects daily practice. These results can provide clinical evidence regarding whether BVA can be beneficial in pain alleviation and changing disease-related functional status.

Previous randomized clinical trials that compared the efficacy of BVA with acupuncture or normal saline injection on LBP [29,30] have been underestimated due to their poor methodological quality. In this study, participants from both the real and sham BVA groups will be treated by the same protocol to minimize the non-specific and placebo effects. All procedures (including the BVA increment protocol administered into predefined acupoints) have been rigorously designed by a process of consensus with experts and previous research according to the Standards for Reporting Interventions in Clinical Trials of Acupuncture. However, the blinding and allocation concealment could be compromised by the manifestation of a bee-venom-specific response in patients in our experimental group, including pain, swelling, redness of the skin and itching, which could be perceived by patients. We will try to blind and conceal group allocation between patients, assessors, acupuncturists and researchers by having an independent researcher prepare both real and sham BVA procedures. The results of this trial will be available in August 2013.

### Trial status

The trial is currently in the recruitment phase.

## Abbreviations

BDI: Beck’s Depression Inventory; BVA: Bee venom acupuncture; CLBP: chronic low back pain; EQ-5D: EuroQol 5-Dimension; KHUHGD: Kyung Hee University Hospital at Gangdong; KMD: Korean medical doctors; LBP: lower back pain; ODI: Oswestry Disability Index; VAS: visual analog scale.

## Competing interests

The authors declare that they have no competing interests.

## Authors’ contributions

BKS is responsible for developing the treatment protocol, carrying out the clinical study and drafting the manuscript. JHL contributed to the study conception and design, participated in the acquisition of data and revised the manuscript. WSS and EMS carried out the clinical study and participated in manuscript drafting. DJJ contributed to the design of the study and supervised the protocol fulfillment and the acquisition of funding for the study. All authors have read, revised and approved the final manuscript.
